# Welfare of Farmed Crocodilians: Identification of Potential Animal-Based Measures Using Elicitation of Expert Opinion

**DOI:** 10.3390/ani11123450

**Published:** 2021-12-03

**Authors:** Leisha Hewitt, Alison Small

**Affiliations:** 1 Roseworthy Campus, School of Animal and Veterinary Sciences, The University of Adelaide, Roseworthy, SA 5371, Australia; 2 CSIRO Agriculture and Food, Armidale, NSW 2350, Australia; Alison.Small@csiro.au

**Keywords:** crocodilian, animal welfare, animal-based measure, animal-based indicator, welfare assessment, welfare measure

## Abstract

**Simple Summary:**

This study focuses on an elicitation of expert opinion to identify a toolbox of animal-based measures that can be used to assess the welfare of farmed crocodilians. This is the initial step towards identifying an animal-based assessment protocol that could be used to support the international outcome-based standard developed by the crocodilian farming industry. Potential measures were identified and aligned with the four animal welfare principles and twelve criteria developed by Welfare Quality^®^, focusing primarily on practical measures that could be used for monitoring farm processes or during external verification activities. The proposed measures were presented to a panel made up of animal welfare specialists (farmers, veterinarians and scientists) for judgment and scoring. Twenty-eight experts scored the proposed measures for validity (that being the relevancy to the welfare criterion and usefulness as a measure) and feasibility (that being how easy it is to observe and assess, for example, during an on-farm animal welfare assessment or routine monitoring). Future studies, involving the preliminary testing of the measures on a commercial crocodile farm, are planned to confirm validity and establish the reliability of the identified measures.

**Abstract:**

Animal-based measures are the measure of choice in animal welfare assessment protocols as they can often be applied completely independently to the housing or production system employed. Although there has been a small body of work on potential animal-based measures for farmed crocodilians, they have not been studied in the context of an animal welfare assessment protocol. Potential animal-based measures that could be used to reflect the welfare state of farmed crocodilians were identified and aligned with the Welfare Quality^®^ principles of good housing, good health, good feeding and appropriate behaviour. A consultation process with a panel of experts was used to evaluate and score the potential measures in terms of validity and feasibility. This resulted in a toolbox of measures being identified for further development and integration into animal welfare assessment on the farm. Animal-based measures related to ‘good feeding’ and ‘good health’ received the highest scores for validity and feasibility by the experts. There was less agreement on the animal-based measures that could be used to reflect ‘appropriate behaviour’. Where no animal-based measures were deemed to reliably reflect a welfare criterion nor be useful as a measure on the farm, additional measures of resources or management were suggested as alternatives. Future work in this area should focus on the reliability of the proposed measures and involve further evaluation of their validity and feasibility as they relate to different species of crocodilian and farming system.

## 1. Introduction

### 1.1. Crocodilian Farming Standards

The farming of crocodilians is a relatively new, though economically important, livestock sector. Farming systems either comprise of closed-cycle captive breeding, independent of the wild populations, ranching, which involves the collection of eggs from the wild to be hatched and raised in the farming system, or a combination of both. Crocodilians are ectothermic vertebrates, belonging to the most ancient order of reptile, Crocodylia. There are 24 species of crocodilians currently recognised, with distinct anatomical, physiological and behavioural differences observed between families. The majority of farmed crocodilians belong to the crocodile family (*Crocodylus porosus* and *Crocodylus nyloticus*) and alligator family (*Alligator mississippiensis* and *Caiman crocodilus*). There are an estimated 5000 crocodilian farms around the world, most being village-level enterprises, with simple facilities and technology. However, most of the global skin production originates from sophisticated farming enterprises, with infrastructure and management systems designed to provide high levels of animal care [1]. As with conventional livestock species, there is an expectation that crocodilians raised for their skins and meat are afforded a good quality of life and a humane death. Consumers are increasingly concerned about animal welfare and expect livestock industries to demonstrate that acceptable standards of production are being consistently achieved. In response to the call for greater transparency, the crocodilian farming industry has developed and implemented an International Standard for Crocodilian Farming [1]. The standard was developed following international guidelines for standard development and contains mandatory requirements covering processes from breeding to slaughter. The focus of the standard is the achievement of acceptable animal welfare outcomes and demonstration of continuous animal welfare improvement. Part of the standard development procedure involves the need to consider an outcome-based approach and where possible the integration of animal-based measures to reflect real animal welfare improvement [2,3]. Although significant advances have been made in crocodilian husbandry practices and there has been a small body of work on potential animal-based measures for farmed crocodilians [4,5,6] there is no validated animal welfare assessment protocol for farmed crocodilians. The shortage of standardised methods to quantify and address reptilian welfare has also been highlighted in studies of other reptile species [7]. To fill this gap and to demonstrate continued animal welfare improvement, it is deemed necessary to develop animal welfare assessment protocols that are both scientifically valid and practical to implement [8]. This study represents the first effort to develop a toolbox of measures, which can be used to support standards of crocodilian farming, contributing to a better understanding of crocodilian welfare and supporting informed public awareness regarding the farming of exotic species.

### 1.2. Animal Welfare Assessment

Animal welfare is a scientific term that describes a measurable quality of a living animal [9]. The assessment of animal welfare should consider an animal’s physical functioning and fitness and mental state [10,11]. It is also often argued that a measure of animal welfare should not only indicate the absence of negative affective states, but also the presence of positive affective states [12,13,14].

**Figure 1:**
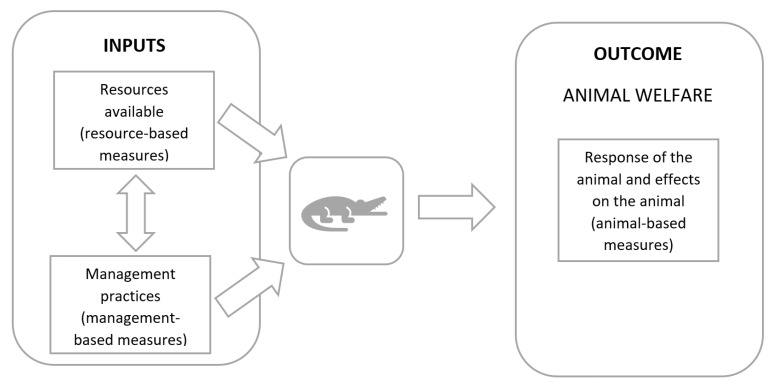
Interplay between resources, management and animal welfare outcome (adapted from EFSA, Bernhard [14]).

The factors that affect an animal’s welfare include its physical environment and the resources available to it (determined using resource-based measures), such as space allowances and housing conditions and the management practices undertaken on the farm (determined using management-based measures), such as provision of pain relief during husbandry procedures, veterinary treatment and animal handling methods. The interplay between available resources and management practices (inputs) and the animal’s welfare state (outcome) is represented in Figure 1 (adapted from EFSA, Bernhard [14]).

**Figure 2:**
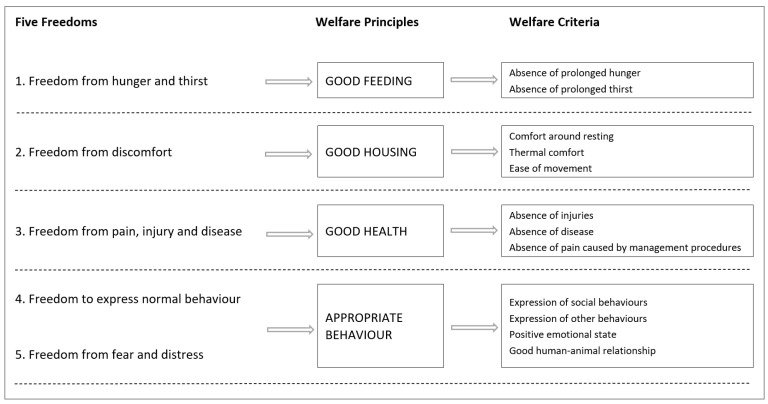
Evolution of the Welfare Quality animal welfare criteria^®^ [18,25].

The relationship between resources, management and the resulting welfare outcome formed the basis of the EU Welfare Quality^®^ project. Welfare Quality^®^ defined four welfare principles, linked to twelve criteria [15], utilising the enduring principles of the Five Freedoms [15,16] (Figure 2). Each principle was developed to identify a key welfare area and is further subdivided into ‘an exhaustive, but concise’ list of criteria [15]. Animal-based measures were chosen by the European Welfare Quality^®^ Project [17] as the preferred tools to assess the actual welfare state of an animal, with protocols initially developed for cattle, pigs and poultry [18]. Subsequently, practical on-farm welfare assessment protocols have been identified and developed for other species, such as horses [19,20], dolphins [21], mink [22], dogs [23] and blue-tongued skink [7]; however, no such protocols currently exist for farmed crocodilians. Use of the Welfare Quality^®^ model does not rely on the use of specialised equipment and can, in theory, be applied to all aspects of the production cycle from hatching through to humane killing. It is important that there is careful selection of the animal-based measures used within an assessment protocol; being considerate of the species and the animal’s environment and being sufficiently repeatable such that different assessors can provide consistent and valid outputs [14]. A perceived downside of the Welfare Quality^®^ protocol is that it can be difficult to apply at farm level, due to the time-consuming assessment process [24]. Therefore, in this study, we aimed to identify meaningful and practical animal-based measures that could be easily integrated into existing animal welfare standards; encouraging uptake and effective use on the farm.

The first step in the development of an animal welfare assessment protocol is the identification of animal-based measures. Animal-based measures are related specifically to the animal, for example, behaviour, body condition and health, whilst resource- and management-based measures cover the provisions associated with animal husbandry, for example, space allowance, feeding regimes and environmental characteristics. Animal-based measures can be broadly categorised as ‘direct’ (e.g., body condition score, observation of lameness, observation of behaviour, etc.) or ‘indirect’ (e.g., records of mortality, feed intake, growth rate, etc.) [18,26]. Usually animal-based measures are recorded at individual level, but once measured they can be aggregated at a group level to be interpreted against defined thresholds of acceptability [14]. EFSA [14] requires animal-based measures to be appropriate; i.e., how well a measure correctly assesses a specific welfare outcome (validity) and how practical it is to perform (feasibility). As observed during the development of the Welfare Quality^®^ protocol, feasibility requires the measures to be quick and simple to record [15] and easy to integrate into the normal farming activities and routine. Measures should also be reliable, meaning that they have low variability when repeatedly used by the same or different observers. The characteristic of reliability was not evaluated as part of this study but will require consideration in the future.

## 2. Materials and Methods

### 2.1. Preparation of Potential Measures

The aim of the study was to identify potential animal-based measures that could cover the whole multidimensional concept of animal welfare (physical and mental state) in crocodilians. There has been very little research focused on the welfare of reptiles [7,27], although some animal-based measures associated with the assessment of other species may be useful for the assessment of crocodilian welfare and were therefore included in the expert elicitation exercise for evaluation.

The Welfare Quality^®^ framework of principles and animal welfare criteria was used to categorise potential animal-based measures. A total of 48 potential measures were aligned with the 12 animal welfare criteria (Table 1) for presentation to the panel of experts for judgement and scoring. The emphasis was on animal-based measures that could be used on the farm; however, additional physiological measures were also included, whilst acknowledging that although the collection of samples could occur on-farm, further laboratory analysis would also be required.

### 2.2. Elicitation of Expert Opinion

The potential animal-based measures were submitted for judgment and scoring to an identified panel. An expert elicitation exercise is considered to be when a recognised expert is asked to use his or her expertise to make an informed judgement, for instance an estimate of something [28]. It is accepted that different experts can give different answers, depending on their field of expertise. Therefore, choosing an appropriate panel of experts can be quite complex and should involve the development of a required expert profile to formalise the process. For this exercise, selected experts had to fulfil at least one of the following general selection criteria: degree in veterinary medicine, at least 10 years’ experience in crocodilian management or research (i.e., physiology, health, behaviour, welfare or production) or at least 10 years animal welfare research experience. A resume or recommendation by another identified expert was required for selection.

The final panel of 80 identified experts included crocodilian farmers, crocodilian and animal welfare scientists and veterinarians. Involving experts from different disciplines is likely to increase the acceptability of the project outcomes and help identify any potential barriers to the application of the toolbox in practice.

Each expert was contacted by letter to outline the purpose of the exercise. The information provided included a definition of animal welfare and animal-based measures, a full list of the identified measures with explanations of their possible on-farm application, and instructions on how to complete the task. Reference material (short explanatory document with definitions and references) was also supplied to support completion of the exercise, and experts were encouraged to contact the researcher if they encountered any problems.

The experts were required to complete 4 tables, each representing a welfare principle: good housing, good feeding, good health and appropriate behaviour. For each of the welfare criteria listed in the table, a set of potential animal-based measures were provided for judgement and scoring. The experts were asked to score each of the proposed animal-based measures for appropriateness using a six-point scale (0 = lowest score and 5 = highest score). According to EFSA [14], the appropriateness of an animal-based measure refers to its validity and feasibility; therefore, the experts had to allocate two scores to each proposed animal-based measure. For validity, the experts were asked to consider how well the proposed measure assesses the associated welfare criterion. For feasibility, the experts were asked to assess how practical it would be to undertake the measurement during a half-day visit to a farm or as part of a farm’s routine monitoring process. The experts were also encouraged to provide context to their score in a comments box. In addition to the measures presented, the experts were given an opportunity to suggest additional measures.

The target population to be considered was defined as farmed crocodilians from hatching to killing (including breeding stock where appropriate). The instructions requested that the experts completed each table in-full, using their personal experiences, scientific knowledge and published data. The time given to complete the task was one month.

### 2.3. Analysis of the Data

All the answers were checked for errors and the data were analysed to calculate the average validity and feasibility scores for each measure. In addition to calculating the mean score for each measure, a percentage of the maximum possible score (%MPS) was calculated, a technique used in a previous study that examined animal-based measures for cattle pigs and laying hens [29]. The overall %MPS (an average of the validity and feasibility %MPS) was used to rank and compare the measures. A high mean score for validity indicates that the measure was regarded by the experts as one which is strongly associated with the welfare criteria. A high mean score for feasibility indicates that the measure was regarded by the experts as one which could be easily undertaken on the farm. Standard deviations were also calculated to indicate the degree of agreement between experts.

## 3. Results

### 3.1. Response to the Exercise

From the 80 experts contacted, 28 returned completed tables representing a 35% response rate. A low response to the exercise was somewhat expected, particularly as it is a very new area of research in crocodilians and the completed responses came from people who felt that they had sufficient knowledge to make a meaningful contribution the exercise. The respondents were made up of experts from all the identified disciplines, without a single group dominating. The farmers who responded represented operations involving *Crocodylus porosus*, *Crocodylus nyloticus*, *Alligator mississippiensis* and *Caiman crocodilus.* All the returned tables were completed in full, with the exception of the ‘Appropriate behaviour’ table, which was only completed by 25/28 experts (89%), the others citing that they did not have the experience to provide a score for some of the measures.

### 3.2. Judgement and Scoring of Proposed Animal-Based Measures

Table 2 provides a summary of the scores for each of the proposed measures presented alongside the animal welfare principles and criteria. The superscript ‘a’ indicates %MPS greater than 80%, whilst the superscript ‘b’ indicates a %MPS less than 80% but greater than 75%. The five highest-ranking measures for validity were all related to the principles of ‘good feeding’ and ‘good health’. Each achieved a %MPS greater than 80%. The use of mortality as a measure of the ‘absence of disease’ had a %MPS for validity of 96% and was awarded a maximum score by 23/28 (82%) of the experts. Similarly, the use of signs of an effective stun/kill as a measure of ‘absence of pain’ during the killing process also achieved a %MPS for validity of 96%, being awarded the maximum score by 26/28 (93%) of the experts.

The use of mortality as a measure of the ‘absence of disease’ was also regarded by the experts to be a practical measure that could be used on the farm. It had a %MPS for feasibility of 92%. The identification of runt animals as a measure of disease also scored highly for feasibility (92%), as it is a condition that is easily recognised on the farm. However, it was awarded a %MPS for validity of less than 70%, with experts recognising that runting in crocodilians is thought to have a multifactorial cause [30].

Respondent agreement (as indicated by the standard deviation of the means for each animal-based measure) was greatest for measures of ‘good health’. The three measures which had the most agreement for validity were all measures of the ‘absence of disease’; being, mortality (sd 0.38), behaviour (sd 0.57) and ocular/nasal discharge (sd 0.61). Respondent agreement was not as consistent for feasibility, indicating a wider range of opinion how easy it would be to perform the proposed measures on the farm. The three measures that had most agreement for feasibility were mortality (sd 0.56), runting (sd 0.56) and obesity/emaciation (sd 0.80).

The experts also reported additional measures which they considered to be useful for all the welfare criteria. The additional measures were an assortment of animal-, management- and resource-based measures (Table 3).

## 4. Discussion

### 4.1. General Findings

Animal-based measures can be broadly categorised into physiological, behavioural and health variables. The highest scoring animal-based measures were related to ‘good health’ and ‘good feeding’. One likely reason for this is that farmers are already familiar with the measurement of production and health and in many cases already implement them successfully on-farm. There was less confidence in the use of physiological indicators. The experts recognised the validity of the use of biomarkers relevant to the welfare criteria, for example, the analysis of stress hormones. However, at present, this type of measure usually involves taking a blood sample, which requires individual animals to be caught and restrained, a process that itself increases stress. This would not meet the criteria of feasibility and thus biomarkers were allocated consistently lower scores than other measures for feasibility. The development and validation of non-invasive techniques, such as faecal analysis for corticosterone metabolites, could be an effective on-farm measure of the future. A preliminary study in Nile crocodiles (*Crocodylus niloticus*) indicated that faecal glucocorticoid metabolites (FGM) could be detected in crocodilian faeces 7–15 days after an adrenocortocotropic hormone (ACTH) stimulation test and that the FGM levels were stable in the faeces for up to 72 h after defecation [31]. Further work is required to understand the impacts of husbandry practices on FGM in farmed crocodilians and allow interpretation of measured levels. An ACTH stimulation test measures the ability of the adrenal glands to respond to ACTH, which itself is released in response to acute stress.

### 4.2. Good Feeding

The animal welfare principle of ‘good feeding’ is made up of the following criteria: absence of prolonged hunger (including appropriate diet) and absence of prolonged thirst. It is achieved by the animal having access to sufficient and nutritionally adequate food and water. Hunger and thirst can occur not only when feed and water is not available, but also when they are not accessible or the quantity and quality do not meet the animals physiological and behavioural needs. All crocodilians are carnivorous, being an apex predator in the wild. As they are ectothermic, they aim to modify their body temperature through behavioural, physiological and biochemical temperature regulation and do not rely on food to generate heat. They possess a high rate of conversion of food into body tissue and in the wild can routinely go for long periods without feeding at all [32]. Additional suggested measures for ‘good feeding’ are listed in Table 3. Experts suggested that the use of animal-based measures could be supplemented with resource- and management-based measures that focus on the available access to feed and water (for example, feed deck size and design) and the quality of the resources (for example, feed composition and nutritional value).

#### 4.2.1. Absence of Prolonged Hunger

For this criterion, the measures that scored highest were Body Condition Score (BCS) (85%) and feed intake (85%), with both measures scoring > 80% MPS for both validity and feasibility. BCS is a recognised robust animal-based measure for evaluating medium to long-term feeding practices in many species [33]. It has been used successfully in some species of crocodilian, though generally only when examining health in wild populations using weight/measurement rather than a visual scale [34,35]. Further work to develop a simple visual scale, appropriate to species, would enable consistent assessment of this measure. It would also be necessary to develop meaningful thresholds for acceptable BCS in crocodilians and identify the welfare implications for each scoring category in different species and ages. In crocodilians, the relationship between the period of feed deprivation and the resulting BCS is not yet fully understood and requires further study. Regarding the feasibility of the measure, it may be difficult in some systems to perform a visual BCS assessment during the normal daily routine as animals may be in the water or under hides. If this is the case, the use of BCS may be limited to times during when animals are handled or done on an ad hoc basis, e.g., when animals are out on dry ground.

The proposed use of a visual weight estimate, as an alternative animal-based measure for this criterion, was questioned by a number of the experts. The general consensus was that the measure is not as valid as BCS, providing no additional benefits over the use of BCS as a measure. The experts’ confidence in the validity of a visual weight estimate for assessing the criterion (69%) was lower than the use of BCS (89%), and it was also considered to be difficult to assess (66%). The difference in scores between the two measures, may be related to the way in which the measures are performed. A visual weight estimate is likely to involve an assessment of the girth of the animal (around the belly), which is known to fluctuate over short periods of time. BCS, on the other hand, focuses on the areas of the body where fat is deposited over time, such as the fullness of the neck and base of the tail, to indicate a longer-term nutritional status.

Feed intake, that is, the willingness of crocodiles to eat a certain amount of food, was also regarded as a valid and feasible measure of this criterion (89%). A measure of feed intake could be achieved by recording the amount fed and any residual amount left after feeding. In a communal pen environment, this will only be possible on a group level, with the added complication of a proportion of the feed being lost in the water. Monitoring feed intake is particularly relevant when making changes to feed type or formulation or as a possible indicator of poor-quality feed. However, note that crocodilians are opportunistic feeders, eating both carrion and fresh food in the wild [36]. The infrequent feeding habits of crocodilians would require feed intake to be measured overtime, using feed records that are aligned with the corresponding daily environmental conditions (for example, temperature).

#### 4.2.2. Absence of Prolonged Thirst

The two proposed animal-based measures for this criterion were visible signs of dehydration and biomarkers (indicating dehydration). Visible signs of dehydration had a %MPS for validity and feasibility of 69% and 56%, respectively. Dehydrated crocodiles are often seen to have sunken eyes, dry mucus membranes, dry and cracked scutes along the tail and depressed temporal fossa. However, the experts noted that these conditions are also observed in diseased crocodilians or those with poor body condition and therefore may not just be reflective of hydration status. They were also concerned that the appearance of visible signs of dehydration often occurs a long time after welfare has been compromised. Biomarkers of dehydration, such as Packed Cell Volume (PCV) were thought to be a valid measure of dehydration; however, as this is an invasive method that requires a blood sample to be taken, the %MPS for feasibility was comparatively low (47%). This could be overcome by collecting blood samples at harvest; however, there still needs to be more research on the significance of this measure and the physiological normal values for crocodilians.

Since no obvious valid and feasible animal-based measure for assessing the absence of prolonged thirst were identified, the use of resource-based measures around the provision of water of an appropriate quality for drinking may be more appropriate. Crocodilians are farmed in enclosures that allow permanent access to water; therefore, the quality of this water for drinking is important. The impact of processes where access to water may not be possible, for example, some types of transport or holding prior to slaughter, requires further study.

### 4.3. Good Housing

The animal welfare principle of good housing is made up of the criteria: comfort around resting, thermal comfort and ease of movement. It is achieved by access to an environment which allows animals to rest, to move in order to access resources and to maintain a preferred body temperature. Comfort around resting and ease of movement can be affected by the size and quality of the space and the number of animals held in the enclosure. An enclosure that appears large may still be insufficient if it cannot successfully service the needs of all the animals held within it. The OIE Terrestrial Animal Health Code [37] states that an animal’s physical environment “should allow comfortable resting, safe and comfortable movement including normal postural changes”. The experts suggested additional resource- and management-based measures to support the use of animal-based measures, for example, space allowance, physical characteristics of the pen (e.g., provision of hides, size of the enclosure), environmental measures (temperature and humidity) and cleanliness of the enclosure.

#### 4.3.1. Physical Comfort around Resting

The experts scored a measure of posture and orientation the highest for this criterion (83%), with the measure scoring %MPS for feasibility of 88% and 78% for validity (overall %MPS of 83%). The measure was thought to reflect the ability of a crocodilian to adopt its preferred postural position within the enclosure, rather than be forced to adopt an unnatural position. The experts also allocated the use of behavioural repertoire an overall %MPS of 75%. This approach, which studies the amount of time that an animal devotes to certain activities (activity time budget) in a fixed period has been used to establish resting comfort in other species [38]. The experts scored behavioural repertoire higher for validity (77%) than for feasibility (72%), possibly reflecting the time-consuming nature of this measure and the current lack of baseline behavioural data for farmed crocodilians.

#### 4.3.2. Thermal Comfort

The experts scored the measure of growth rate the highest for this criterion (78%), with the measure scoring %MPS of 79% for feasibility and 77% for validity. The growth rate of juvenile crocodiles is closely related to the temperature of their environment, with the fastest growth rates occurring in individuals kept in their preferred temperature range [39,40,41]. Behavioural signs of overheating and chilling were scored lower than expected by the experts, despite being useful measures in other species [19,23,42]. This is likely to be related to the fact that crocodilians are ectotherms, their body temperature depending on their environment and not on heat produced metabolically as in mammals and birds. Through behavioural, physiological and biochemical adjustments, crocodilians are able to modify their body temperature to avoid being too hot or too cold [43]. Therefore, it is normal to see wider ranges of body temperature in crocodilians, without any obvious behavioural signs of overheating or chilling.

In the wild, crocodilians usually have the opportunity to select a different environment when ambient temperatures deviate from their preferred ranges. When farmed, it is necessary to either provide crocodilians with their preferred conditions (through effective environmental control) or provide them with a choice of environmental conditions (for example, including materials with different thermal properties), such that they can behaviourally regulate temperature (i.e., move between warmer and colder areas). Demonstrating that crocodilians have access to appropriate thermal conditions, as indicated by measurements of environmental temperature (for example, floor, water and ambient temperatures), was suggested by 10/28 (36%) of the experts. Non-contact infrared thermometers could potentially be used to measure skin temperature as an alternative to cloacal measures of temperature. This makes the measure of body temperature more feasible, as it removes the need to individually capture and restrain crocodilians to take the measurement. However, the relationship between skin temperature and core body temperature still needs to be established for crocodilians.

#### 4.3.3. Ease of Movement

Welfare Quality^®^ describes the criteria of ‘ease of movement’ as the animal having enough space in which to move around. As also noted for the criterion of ‘physical comfort during resting’, observation of the behavioural repertoire was scored quite highly for ‘ease of movement’, being allocated an overall %MPS of 78%. Although time consuming and quite difficult in some housing systems, an activity time budget approach would be useful as a measure of this criterion. In some species of farm and zoo animals, the presence of locomotory stereotypies has been identified as a possible animal-based measure due to the association with restricted space [44,45]. The panel of experts was not confident in the use of locomotor stereotypies for crocodilians, with some experts citing that this type of abnormal behaviour has not been witnessed or reported. Experts also suggested that the animal-based measures for this criterion should be supported by resource-based measures such as the size of the enclosure, where a minimum size must be provided which allows ease of movement.

### 4.4. Good Health

The animal welfare principle of good health is made up of the criteria: absence of injuries; absence of disease; and absence of pain induced by management procedures. Animal-based measures of injury and disease have good face validity, since most injuries and disease are associated with compromised animal welfare, through the experience of pain and distress. Wounds may be sustained due to interactions (aggressive and non-aggressive) with other animals [46] or through the design and maintenance of the enclosure. Overt signs of disease and injury can usually be assessed reliably, though other more subtle signs may remain undetected.

#### 4.4.1. Absence of Injuries

For this criterion, the measures which scored highest were measures of wounds, lameness and skin quality, with all three being allocated an overall %MPS greater than 80%. Measures of wounds and skin quality are probably best performed during harvesting processes; however, wounds on the visible parts of body could also be spotted during routine farming activities. Measurement of skin quality after processing scored an overall %MPS of 80%. Although the experts considered it to be a valid and feasible measure, the nature of the grading process would result in even superficial scratches being recorded, i.e., important for quality, but highly unlikely to be reflective of an animal welfare issue.

#### 4.4.2. Absence of Disease

For this criterion, the measures which scored the highest overall %MPS were mortality (94%) and other physical symptoms of disease, such as ocular/nasal discharge (84%), runting (80%) and deformities (77%). Behaviour, reflective of the negative affective experiences associated with disease, was also scored highly (83%). Skin quality scored a similar overall %MPS for this criterion (81%) as it did for absence of injuries (80%). Mortality is a useful animal-based measure of disease [37]. As a single measure of welfare, it is often regarded as insufficient [33]; however, its usefulness and value during monitoring and intervention cannot be overlooked. The experts also suggested additional management-based measures for this criterion related to access to and use of veterinary treatment and routine antibiotic use.

#### 4.4.3. Absence of Pain Induced by Management Procedures

Absence of pain induced by management procedures is an animal welfare criterion related to any specific husbandry practices that could potentially cause the animal pain. This would include any methods used for euthanasia or during the commercial killing process. For some species of crocodilian, this criterion could also include the application of electrical stunning, which is sometimes used for capture and restraint.

The measures that scored highest overall %MPS for this criterion were signs of an effective stun/kill (91%) and physical damage (84%). Signs of effective stunning and killing have recently been reviewed in chapter 7.14 of the OIE Terrestrial Animal Health Code—Killing of reptiles for their skins, meat and other products [6]. The OIE describes the use of animal-based measures for the verification of unconsciousness or death, with the presence of any of the following criteria being sufficient to establish suspicion of consciousness in crocodilians: pupillary response to light or moving objects, eye movement in response to objects or movement, blink or nictitating membrane responses to touch or contact of the cornea in species where eyelids are present, spontaneous eyelid opening or closing, intentional defensive responses and tongue movement. For any other husbandry procedures likely to cause pain, the expert panel suggested that a useful management-based measure could be the use of an effective pain management protocol, involving anaesthesia and analgesia.

### 4.5. Appropriate Behaviour

The animal welfare principle of appropriate behaviour is made up of the criteria: expression of social behaviours, expression of other behaviours, positive emotional state and good human–animal relationships. This principle considers that animals should be able to express normal, non-harmful and important behaviours [18]. Change in an animals’ behaviour is often the first and most obvious indicator of its ability to cope with its environment [47]. However, there is a paucity of information relating to both field-based observations of reptilian behaviour [7] and behaviour as it relates to the welfare of reptiles [27]. Crocodilian behaviours can be subtle and often difficult to interpret [48,49], with individuals spending significant periods of time in an apparent motionless state, watching but not moving [50]. Relatively little is known about their social behaviours in the context of a farming environment.

#### 4.5.1. Expression of Social Behaviours

This criterion recognises that for some species, the presence of conspecifics may facilitate positive social interactions, also called affiliative behaviours. Affiliative behaviour is readily observed in the ‘more social’ species [51,52,53,54]; however, the significance of affiliative behaviours in crocodilians is certainly very under-researched. Cooperative feeding has been described in *Crocodylus nyloticus*, *Alligator mississippiensis* and *Caiman crocodilus* [55], in which individuals apparently work together to capture prey; however, it is somewhat unclear whether these are simply ‘feeding aggregations’ rather than examples of true affiliative behaviour. Despite this lack of clarity, it is certain that some cooperation can be observed, as indicated by the tolerance of individuals being in close proximity to one another. Crocodilians are certainly not considered to be highly social animals, with interspecific competition (sometimes resulting in mortality) and even cannibalism observed in wild populations [56,57]. Two measures of this criterion were therefore suggested to the expert panel: co-occupant aggression and affiliative behaviour. The scoring and feedback from the expert panel was that affiliative behaviour and its usefulness as a measure in crocodilians is not yet sufficiently understood (overall %MPS of 71%), this was further reflected by three experts declining to score this measure, citing lack of knowledge in this area as the reason. Co-occupant aggression was scored slightly higher with an overall %MPS of 73%, with some experts suggesting that this measure could be used alongside resource-based measures of space allowance, access to feed (e.g., feed deck space and arrangement) and group size.

#### 4.5.2. Expression of Other Behaviours

The criterion of ‘expression of other behaviours’ is related to the opportunity for animals to perform important behaviours, as well as the identification of abnormal or undesirable behaviours. Behaviours observed in the wild are often related to sexual maturity and competition for resources. This means that they are often driven by stimuli that are not usually present in a captive environment, due to the age of the farmed animals and sufficient access to resources. The presence of abnormal behaviours in animals, i.e., those that are not present in wild counterparts and serve no obvious function [58], may indicate sub-optimal welfare [59]. Abnormal behaviours are usually described as ‘vacuum behaviours’, developing when an animal is prevented from doing a behaviour that they are motivated to perform. The absence of abnormal behaviours was considered by the experts to be a potentially useful measure of this criterion in crocodilians (overall %MPS of 78%). Specific examples of abnormal behaviours that could be present included piling and stargazing. The group response to low-grade stimuli, for example walking around the pen and the distribution of animals throughout the pen were also suggested as measures.

#### 4.5.3. Positive Emotional State

Research into conditions which have a negative impact on an animal’s affective state far outweigh the studies on positive affective state, particularly in reptiles [7,27]. For other reptile species, literature on positive welfare state is almost entirely focused on the provision of enrichment [7]. Effective enrichment is the provision of conditions that allow an animal to undertake highly motivated behaviours, rather than something designed to provide variety or novelty. The role of enrichment and its value for farmed crocodilians has not been determined.

There is also a growing body of evidence that suggests that behavioural diversity may be a potential positive indicator of animal welfare [60]. Behavioural diversity can be defined as the frequency and richness of species-typical behaviour exhibited by an individual animal [61]. This has been studied in cheetahs [61] and dolphins [62], where in both studies there was an inverse relationship between faecal cortisol metabolites and behavioural diversity, supporting the idea that behavioural diversity may contribute to a more positive emotional state. Whilst behavioural diversity has not been validated as an indicator of a positive emotional state, further studies in this area would be beneficial. The animal-based measure of behavioural repertoire scored an overall %MPS of 75%. The experts scored this measure highly for validity (%MPS of 81%). It scored much lower for feasibility (%MPS of 69%), which is likely to reflect some of the previously identified issues around interpretation of crocodilian behaviour.

A useful initial step would be to develop a behavioural ethogram and activity time budget of farmed crocodilians. It was suggested by the experts that video surveillance may be a useful tool for the periodic assessment of behavioural repertoire.

#### 4.5.4. Good Human–Animal Relationships

This criterion is related to the appropriateness of animal handling, where animal handlers are required to promote a ‘good human–animal relationship’ to achieve a good animal welfare outcome [63]. Signs of human-directed aggression are not uncommon in crocodilians and a close human–animal relationship between handler and crocodilian is unlikely. Despite this, it is still expected that stock people treat crocodilians in a positive and compassionate manner by ensuring that any interactions are performed quietly, calmly and using controlled movements. It is doubtful that crocodilians will ever reach the level of domestication seen in other livestock species, and handling is always likely to evoke a fear response. Experts considered that an assessment of animal behaviour in response to the animal handler could be feasible (%MPS for feasibility of 75%) using observations of behaviours such as piling and distress calls.

Reducing exposure to and duration of handling is another means by which a good human–animal relationship could be achieved in crocodilians. Other measures suggested by the experts were focused on the knowledge and skill of the stockperson and included use of appropriate husbandry and handling methods, training and competency, adherence to routine (to increase habituation and reduce panic behaviour) and attitude of the stockperson. The OIE [37] recommends that reptile handlers should be competent in handling, moving, stunning and verifying effective stunning and killing, as well as understanding species-specific behaviours and the underlying animal welfare and technical principles necessary to carry out the tasks. The OIE also recommends that handling, restraining, stunning and killing should take into account the following characteristics of reptiles: sensitivity and responsiveness to visual, tactile, auditory, olfactory and vibrational stimuli; ability to escape handling and restraint because of their agility and strength; ability to inflict significant injuries to handlers; slow movements, torpor and reduced responsiveness due to low body temperatures or slow metabolic rates, which should not be regarded as indicators of quiescence or unconsciousness; and the absence of vocalisation, which is typical in reptiles, even in highly traumatic situations.

### 4.6. Toolbox of Animal Welfare Outcome Measures

The overall objective of this study was to create a toolbox of valid and feasible animal-based measures that could be used during an animal welfare assessment of farmed crocodilians (Table 4). It focused on the critical aspects of crocodilian farming that could negatively impact crocodilian welfare, as indicated by the Five Freedoms paradigm [25] and aligned with the principles and criteria of Welfare Quality^®^. The toolbox applied a multifaceted approach, including several indicators for each welfare criterion where possible. It recognises the interplay between input and output measures, where animal-based measures (the output) can be used to validate the appropriateness of the resources used [64] and where resource- and management-based measures can also be used in the absence of validated animal-based indicators.

All invasive measures or those that involved specific handling of the crocodilian for the purpose of taking the measurement were excluded from the toolbox; however, their usefulness on-farm after certain refinements warrants further investigation.

The identified measures incorporated into the toolbox possess content validity, that is, they have been identified through a review of scientific literature and further validated by a panel of experts [29]. This study did not aim to quantify the extent of the welfare impact. For instance, concerning animal health, to what extent do wounds and abrasions affect the welfare outcome? Future studies should be undertaken to provide us with a greater insight into this issue and further investigate the complex interactions between animal-based measures and resource and management conditions. In comparison to other livestock species, there is still a lack of information regarding the relevance of negative states in crocodilians, so more research is needed in this area. Additional research should also attempt to understand more about crocodilian behavioural repertoire and determine the relevance of positive experiences to crocodilians, for example, the significance of environmental enrichment and behavioural diversity.

The main concern regarding welfare assessment is the extent to which we are measuring what we are supposed to be measuring. As used in this study, an elicitation of expert opinion is one recognised method of assessing the validity and feasibility of animal-welfare measures [14]. During this study, it was deemed essential that veterinary, crocodilian or animal welfare expertise was thoroughly integrated into the assessment of each measure. This was achieved by making it a prerequisite for the expert panel. Despite this, it would still be beneficial to conduct further validation trials to fully understand the relationship between specific welfare outcome measures and the welfare criteria they are designed to address and to assess reliability. Furthermore, a number of gaps in knowledge were identified during the process of this expert elicitation.

The adoption of animal-based measures is also dependent on the ability to perform them easily, quickly and repeatably [15]. It is envisaged that the primary purpose of the toolbox will be on-farm monitoring and assessment; therefore, it is important that the measures can be undertaken using non-invasive techniques and in an efficient manner. Selected measures can be applied practically and undertaken as part of the normal crocodilian farming activities.

## 5. Conclusions

It is intended that the toolbox be further developed for several purposes, such as the evaluation of known animal welfare hazards, monitoring the success of any husbandry interventions used on the farm, assessing the impact of different production and management conditions and benchmarking current performance of a farm (to monitor future improvements or for comparison with other farms). This is the first step to quantifying and systematically measuring welfare in farmed crocodilians. The outcome of the project can be used to continuously improve industry practice, providing a basis for outcome-based certification standards and supporting informed public awareness regarding the farming of crocodilians.

## Figures and Tables

**Table 1:** Alignment of the potential animal-based measures with the 12 welfare criteria of the Welfare Quality^®^ assessment protocol.

Welfare Principle	Welfare Criteria	Proposed Measures ^1^
Good feeding	Absence of prolonged hunger	Body condition score, visual weight estimate, food seeking behaviour, feed intake, stomach contents, growth rate, normal faecal mass
	Absence of prolonged thirst	Visible indicators of dehydration, biomarkers indicative of dehydration
Good housing	Physical comfort around resting	Posture and orientation, behavioural indicators, skin quality, stress biomarkers,
	Thermal comfort	Posture and orientation, behaviour (signs of overheating or chilling), skin quality, growth rate, body temperature
	Ease of movement	Behavioural indicators (caused by a restrictive environment), locomotor stereotypies
Good health	Absence of injuries	Wounds, skin quality, lameness, abrasions
	Absence of disease	Mortalities, deformities, presence of ocular or nasal discharge, behavioural indicators, skin quality, presence of parasites, respiration rate, runting, body position
	Absence of pain induced by management procedures	Physical damage, behavioural signs of ineffective stunning and killing, physical movement, stress biomarkers
Appropriate behaviour	Expression of social behaviours	Affiliative behaviour (social cohesion), co-occupant aggression
	Good animal–human relations	Human-directed aggression, behavioural indicators (reflective of human–animal interaction)
	Positive emotional state	Stress biomarkers, behavioural indicators, obesity and emaciation
	Expression of other behaviours	Absence of abnormal behaviours

^1^ Participants were also provided with the opportunity to suggest additional measures.

**Table 2:** Individual animal-based measures ranked according to mean validity score (0 = low, 5 = high): highest mean score considered to be the most valid for measuring the welfare criteria.

Welfare Principle and Criteria	Animal-Based Measure	Validity		Feasibility		Overall %MPS
		Mean	% MPS	Mean	% MPS	
Good feeding: Absence from prolonged hunger	Body condition score	4.4 ± 0.68	89 ^a^	4.0 ± 1.15	81 ^a^	85 ^a^
	Feed intake	4.3 ± 0.76	86 ^a^	4.2 ± 0.89	84 ^a^	85 ^a^
	Growth rate	4.2 ± 0.87	85 ^a^	4.0 ± 1.12	79 ^b^	82 ^a^
	Visual weight estimate	3.4 ± 0.94	69	3.3 ± 1.28	66	68
	Food seeking behaviour	2.8 ± 1.31	56	2.3 ± 1.14	46	51
	Stomach contents	1.9 ± 1.61	38	2.2 ± 1.88	44	41
	Faecal mass	2.5 ± 1.27	50	1.5 ± 1.43	30	40
Good feeding: Absence from prolonged thirst	Visible signs dehydration	3.5 ± 1.32	69	2.8 ± 1.31	56	63
	Biomarkers of dehydration	3.6 ± 1.23	73	2.4 ± 1.49	47	60
Good housing: Physical comfort around resting	Posture and orientation	3.9 ± 1.24	78 ᵇ	4.4 ± 0.94	88 ᵃ	83 ᵃ
	Behavioural repertoire	3.9 ± 1.13	77 ᵇ	3.6 ± 1.35	72	75 ᵇ
	Skin quality	3.3 ± 1.15	65	3.6 ± 1.27	71	68
	Stress biomarkers	3.3 ± 1.06	65	2.8 ± 1.51	56	61
Good housing: Thermal comfort	Growth rate	4.0 ± 0.98	79 ᵇ	3.9 ± 1.16	77 ᵇ	78 ᵇ
	Behaviour-overheating	3.8 ± 1.05	76	3.3 ± 0.99	65	70
	Skin quality	3.2 ± 1.40	64	3.7 ± 1.13	74	69
	Behaviour-chilling	3.5 ± 1.43	69	2.9 ± 1.19	57	63
	Body temperature	3.1 ± 1.46	63	2.4 ± 1.47	49	56
Good housing: Ease of movement	Behavioural repertoire	4.1 ± 1.22	81 ᵃ	3.9 ± 1.44	74 ᵇ	78 ᵇ
	Locomotor stereotypies	3.0 ± 1.55	61	2.5 ± 1.72	50	55
Good health: Absence of injuries	Wounds	4.3 ± 0.76	86 ᵃ	3.9 ± 1.06	77 ᵇ	82 ^a^
	Lameness	4.0 ± 1.02	81 ᵃ	4.0 ± 0.82	81 ᵃ	81 ^a^
	Skin quality	4.1 ± 0.80	81 ᵃ	4.0 ± 0.87	79 ᵇ	80 ^a^
	Abrasions	4.1 ± 0.91	83 ᵃ	3.8 ± 0.95	75 ᵇ	79 ᵇ
Good health: Absence of disease	Mortality	4.8 ± 0.38	96 ᵃ	4.6 ± 0.56	92 ᵃ	94 ᵃ
	Ocular/nasal discharge	4.4 ± 0.61	87 ᵃ	4.1 ± 0.92	81 ᵃ	84 ᵃ
	Behaviour	4.5 ± 0.57	89 ᵃ	3.8 ± 1.18	76 ᵇ	83 ᵃ
	Skin quality	4.0 ± 0.98	81 ᵃ	4.1 ± 0.90	82 ᵃ	81 ᵃ
	Runting	3.4 ± 1.02	69	4.6 ± 0.56	92 ᵃ	80 ᵃ
	Deformities	3.5 ± 1.30	70	4.2 ± 1.11	84 ᵃ	77 ᵇ
	Position of the body	2.6 ± 1.05	52	3.5 ± 1.16	70	61
	Presence of parasites	3.0 ± 1.22	6	2.7 ± 1.26	54	57
	Respiration rate	3.1 ± 1.57	62	2.3 ± 1.73	46	54
Good health: Absence of pain induced by management procedures	Signs of an effective stun/kill	4.8 ± 0.66	96 ᵃ	4.3 ± 0.93	86 ᵃ	91 ᵃ
	Physical damage	4.4 ± 0.86	89 ᵃ	4.0 ± 0.91	79 ᵇ	84 ᵃ
	Physical movement	3.8 ± 1.01	76 ᵇ	4.0 ± 1.13	80 ᵃ	78 ᵇ
	Stress biomarkers	3.6 ± 1.15	71	2.5 ± 1.45	51	61
	Vocalisation	2.7 ± 1.44	54	3.1 ± 1.75	61	58
Appropriate behaviour: Expression ofsocial behaviours	Co-occupant aggression	3.8 ± 0.86	76 ᵇ	3.5 ± 1.17	70	73
	Affiliative behaviour	3.5 ± 0.96	71	3.6 ± 0.91	72	71
Appropriate behaviour: Good animal–human relation	Behaviour	3.7 ± 1.07	74	3.8 ± 1.09	75 ᵇ	74
	Human-directed aggression	3.4 ± 1.49	67	3.8 ± 1.32	76	71
Appropriate behaviour: Positiveemotional state	Obesity/emaciation	4.1 ± 0.94	82 ᵃ	4.3 ± 0.80	86 ᵃ	84 ᵃ
	Behavioural repertoire	4.0 ± 1.20	81 ᵃ	3.4 ± 1.31	69	75 ᵇ
	Stress biomarkers	3.6 ± 1.21	72	2.6 ± 1.47	52	62
Appropriate behaviour: Other behaviours	Abnormal behaviours	4.0 ± 1.16	80 ᵃ	3.8 ± 1.21	76 ᵇ	78 ᵇ

^a^ indicates %MPS greater than 80%, ^b^ indicates %MPS less than 80% but greater than 75%.

**Table 3:** Additional measures proposed for each of the animal welfare criteria by the experts.

Welfare Principle	Animal-Based Measures	Resource-Based Measures	Management-Based Measures
Good feeding	*Postmortem* examination, post-feeding behaviour, skin quality (presence of wrinkles), competition for feed, loose skin	Composition of feed, feed quality, water quality, availability of water (of appropriate quality for drinking), feed deck space and arrangement	Feeding regimen, feed preparation protocol
Good housing	Faecal consistency, pressure sores and abrasions, social dominance	Space allowance, physical characteristics of the pen (e.g., provision of hides, size of the enclosure), environmental measures (temperature and humidity)	Cleanliness and maintenance of the enclosure, adaptation period
Good health	Feed intake, time taken to return to feeding		Access to and use of veterinary treatment, antibiotic used, use of anaesthetic and analgesia for husbandry procedures
Appropriate behaviour	Skin quality	CCTV use, enrichment provision, space allowance, feed deck space and arrangement, group size	Husbandry and handling methods, training and competency, attitude of stockperson

**Table 4:** Toolbox of measures.

Welfare Principle	Welfare Criteria	Suggested Animal-Based Measure	Supporting Resource-Based Measure
Good feeding	Absence of prolonged hunger	Body condition score, feed intake	Distribution of feed, feeding frequency
	Appropriate diet	Growth rate, feed intake	Feed composition and quality
	Absence of prolonged thirst	Lack of animal-based measure	Access to drinking water
Good housing	Physical comfort when resting	Posture and orientation, behavioural indicators	Space allowance, pen design
	Thermal comfort	Posture and orientation, behavioural repertoire	Provision of appropriate thermoregulatory resources, air quality
	Ease of movement	Behavioural indicators	Space allowance, pen design
Good health	Absence of injuries	Wounds, skin quality	Veterinary treatment records
	Absence of disease	Mortality, ocular/nasal discharge, skin quality, behaviour	Veterinary treatment, antibiotic use
	Absence of pain induced by management procedures	Physical damage, signs of an effective stun/kill	Pain management, operator competency
Appropriate behaviour	Expression of social behaviours	Lack of animal-based measures	Access to resources, appropriate grouping for animal type
	Good human–animal relationships	Lack of animal-based measures	Competency of handler
	Positive emotional state	Behavioural repertoire, obesity/emaciation	Access to resources
	Expression of other behaviours	Absence of abnormal behaviours	Access to resources

## Data Availability

Raw data are held as confidential material by Leisha Hewitt, with the permission of the International Crocodilian Farmers Association.
